# Progression of White Matter Disease and Cortical Thinning Are Not Related in Older Community-Dwelling Subjects

**DOI:** 10.1161/STROKEAHA.115.011229

**Published:** 2016-01-25

**Authors:** David Alexander Dickie, Sherif Karama, Stuart J. Ritchie, Simon R. Cox, Eleni Sakka, Natalie A. Royle, Benjamin S. Aribisala, Maria Valdés Hernández, Susana Muñoz Maniega, Alison Pattie, Janie Corley, John M. Starr, Mark E. Bastin, Alan C. Evans, Ian J. Deary, Joanna M. Wardlaw

**Affiliations:** From the Brain Research Imaging Centre (D.A.D., E.S., N.A.R., B.S.A., M.V.H., S.M.M., M.E.B., J.M.W.), Neuroimaging Sciences, Centre for Clinical Brain Sciences (D.A.D., E.S., N.A.R., B.S.A., M.V.H., S.M.M., M.E.B., J.M.W.), Department of Psychology (S.J.R., S.R.C., A.P., J.C., I.J.D.), and Centre for Cognitive Ageing and Cognitive Epidemiology (S.J.R., S.R.C., J.M.S., M.E.B., I.J.D., J.M.W.), Alzheimer Scotland Dementia Research Centre (J.M.S.), The University of Edinburgh, Edinburgh, UK; Scottish Imaging Network, a Platform for Scientific Excellence (SINAPSE) Collaboration (D.A.D., E.S., N.A.R., B.S.A., M.V.H., S.M.M., M.E.B., J.M.W.); Department of Neurology and Neurosurgery, McConnell Brain Imaging Center, Montreal Neurological Institute, McGill University, Montreal, QC, Canada (S.K., A.C.E.); and Department of Psychiatry, Douglas Mental Health University Institute, McGill University, Verdun, QC, Canada (S.K.).

**Keywords:** ageing, brain, cortex, MRI, white matter hyperintensities

## Abstract

**Background and Purpose—:**

We assessed cross-sectional and longitudinal relationships between whole brain white matter hyperintensity (WMH) volume and regional cortical thickness.

**Methods—:**

We measured WMH volume and regional cortical thickness on magnetic resonance imaging at ≈73 and ≈76 years in 351 community-dwelling subjects from the Lothian Birth Cohort 1936. We used multiple linear regression to calculate cross-sectional and longitudinal associations between regional cortical thickness and WMH volume controlling for age, sex, Mini Mental State Examination, education, intelligence quotient at age 11, and vascular risk factors.

**Results—:**

We found cross-sectional associations between WMH volume and cortical thickness within and surrounding the Sylvian fissure at 73 and 76 years (rho=−0.276, Q=0.004). However, we found no significant longitudinal associations between (1) baseline WMH volume and change in cortical thickness; (2) baseline cortical thickness and change in WMH volume; or (3) change in WMH volume and change in cortical thickness.

**Conclusions—:**

Our results show that WMH volume and cortical thinning both worsen with age and are associated cross-sectionally within and surrounding the Sylvian fissure. However, changes in WMH volume and cortical thinning from 73 to 76 years are not associated longitudinally in these relatively healthy older subjects. The underlying cause(s) of WMH growth and cortical thinning have yet to be fully determined.

Brain white matter hyperintensity (WMH) growth and cortical thinning are commonly seen on magnetic resonance imaging (MRI) in community-dwelling older people.^1–5^ The incidence of these features is highly variable between individuals but those with the largest WMH volumes and/or thinnest cortices are at increased risk of stroke, dementia, and cognitive and physical impairment.^6–8^ Effective interventions are dependent on understanding the mechanisms of WMH growth and cortical thinning and whether one feature may be an underlying cause of the other.

Cross-sectional studies have found associations between larger whole brain WMH volume and reduced gray matter (GM) volume, density, and thickness.^3,5,9–12^ Those with larger WMH volumes generally had relatively reduced cortical thickness^3^ and density^5,11^ in frontotemporal and inferior parietal regions. Others have found similar cross-sectional patterns of negative associations between whole brain WMH volume and regional GM volume in the default mode network (which includes medial temporal lobe structures, the inferior parietal lobe, and cuneus) using a region of interest analysis.^10^ Larger WMH volume in small vessel disease patients has also been associated with reduced structural connectivity in frontotemporal and inferior parietal regions.^7^

These studies were cross-sectional and so cannot ascertain a direction of causation or effect. Additionally, the regions of cortical thinning that were associated with WMH volume did not overlie regions with the greatest incidence of WMH,^4^ for example, centrifugally around the ventricles and superiorly towards the cranial vertex. Longitudinal studies are required to determine whether larger WMH volumes at baseline and larger increases in WMH volume are associated with subsequent regional cortical thinning. A longitudinal study of the association between cortical morphology and WMH volume growth has previously been conducted in Cerebral Autosomal Dominant Arteriopathy With Subcortical Infarcts and Leucoencephalopathy (CADASIL) patients.^13^ This study found that although lacunar lesions were strongly related to worsening cortical morphology, WMH volume was not strongly related to worsening cortical morphology in CADASIL.

In the present study, we assessed longitudinal associations between change in WMH volume and change in cortical thickness to determine if the relationship between WMH and regional cortical thinning could be causal in community-dwelling subjects from ages 73 to 76.^3,11^ If the relationship between WMH and the regions of cortex that were thinned was potentially causal, then we hypothesized that there would be an association between (1) baseline whole WMH volume and change in cortical thickness over the next few years; (2) baseline cortical thickness and change in WMH volume; and (3) change in WMH volume and change in cortical thickness. To test these hypotheses, we measured progression of WMH volume and cortical thinning from ≈73 to ≈76 years of age in community-dwelling subjects from the Lothian Birth Cohort 1936 (LBC1936) study.

## Methods

### Study Approval and Subject Consent

Approval for the LBC1936 study protocol was obtained from the Multicentre Research Ethics Committee for Scotland (MREC/01/0/56) and Lothian Research Ethics Committee (LREC/2003/2/29). All subjects gave written, informed consent.

### Subjects

**Figure 1.**
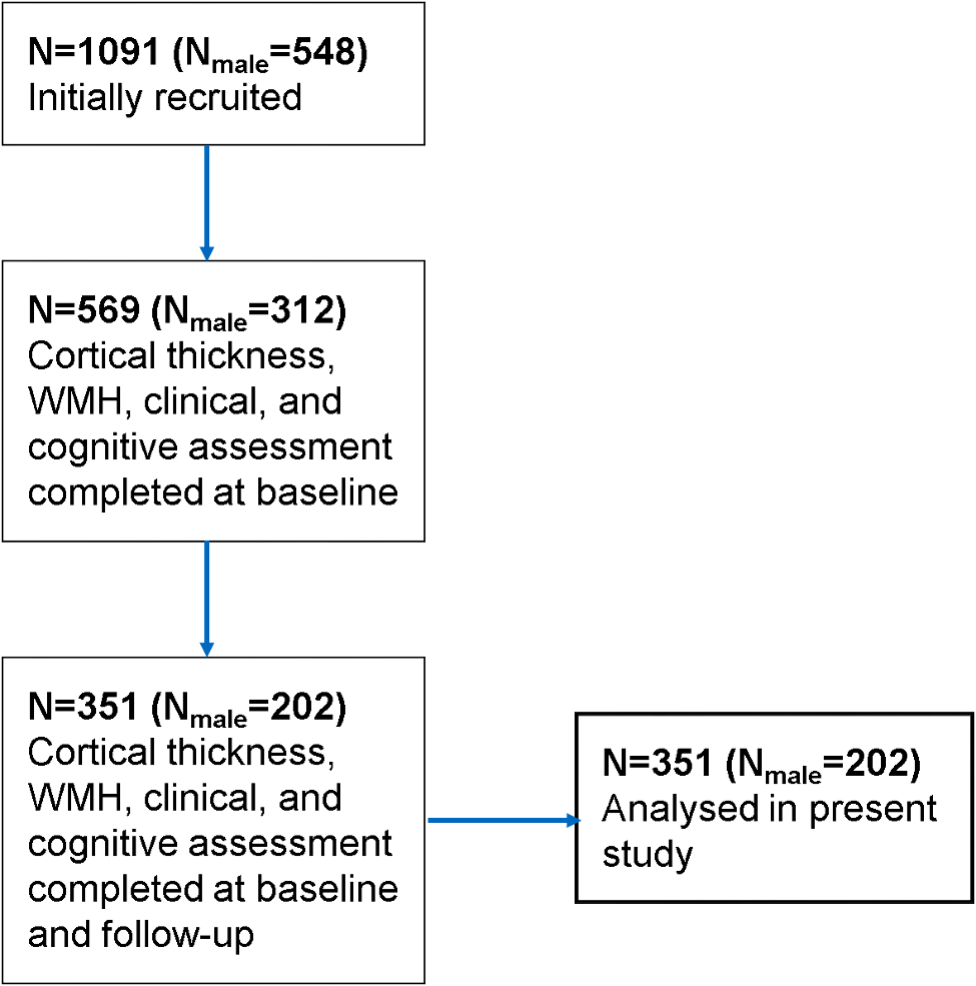
Subject recruitment flow chart. WMH indicates white matter hyperintensity.

In the present study, we assessed 351 (N_male_=202) community-dwelling subjects from the LBC1936 study^14,15^ that had full brain MRI measures, clinical and cognitive assessments at imaging baseline (mean age 72.71±0.72 years), and follow-up (mean age 76.40±0.64 years). These subjects were not deliberately selected, rather they were simply those who agreed to participate in brain scanning and had complete data sets at baseline and follow-up (Figure 1).

We recorded Mini Mental State Examination (MMSE) scores at baseline and follow-up to screen for possible dementia.^14,15^ We did not exclude subjects based on MMSE but included MMSE as an adjustment variable. We recorded the following vascular risk factors (VRF) during a clinical research facility visit: history of hypertension, hypercholesterolemia, diabetes mellitus, smoking, history of cardiovascular disease, and body mass index. History of cardiovascular disease includes self-reported incidences of coronary heart disease, stroke, peripheral arterial disease, and aortic disease. We also took blood samples and measured blood pressure but did not include these variables (eg, systolic blood pressure and glycated hemoglobin) in the present analysis to maximize the number of subjects with complete data sets and to be consistent with previous work.^3^ Further, we have previously shown that historical variables, for example, history of hypertension, have greater associations with WMH than measured variables, for example, systolic blood pressure.^4^

### Brain MRI Acquisition

Brain MRI acquisition parameters were described in detail previously.^16^ Briefly, all subjects had brain MRI on the same 1.5 tesla GE Signa Horizon HDx clinical scanner (General Electric, Milwaukee, WI), maintained on a careful quality assurance programme, at baseline and follow-up. The scanning protocol was the same at baseline and follow-up and acquired T1-, T2-, T2*-, and fluid-attenuated inversion recovery–weighted images.^16^

### WMH Volume and Cortical Thickness Measurement

We measured intracranial volume and whole WMH volume in milliliters using a validated multispectral image processing method that combines T1-, T2-, T2*-, and fluid-attenuated inversion recovery–weighted MRI sequences for segmentation.^17–19^

We measured cortical thickness using the fully automated Civet image processing pipeline developed at the Montreal Neurological Institute.^20,21^ Civet measures cortical thickness at 81 924 vertices (the perpendicular distance between GM and WM surfaces) across the cortex.^20–23^ For clarity, we refer to vertex as the perpendicular distance between GM and WM surfaces, not the cranial vertex.

We manually verified WMH volume masks and cortical thickness maps as per procedures described previously.^17–19,22,23^ Specifically, we followed STRIVE (Standards for Reporting Vascular Changes on Neuroimaging) guidelines for segmenting WMH, manually removing cortical and subcortical infarcts from WMH masks.^19^ Finally, the reliability of cortical thickness^20–22^ and WMH^17–19^ measurements were tested and reported previously.

### Statistical Analysis

All statistical analyses were performed in Matrix Laboratory (MATLAB) R2014a (© 1994–2014 The MathWorks, Inc). We assessed changes in overall mean cortical thickness (mean thickness of the whole cortical mantle), whole brain WMH volume, and continuous variables used in adjustment, for example, body mass index, from 73 years to 76 years using paired *t*-tests. Log-transforming the positively skewed WMH distributions had little effect on our results, and therefore, we maintained their original scale (proportion of intracranial volume) to simplify interpretation. We assessed changes in binary variables used in adjustment, for example, history of hypertension, using *z*-tests of proportion.

Cortical vertex-wise regression analyses were performed using the SurfStat MATLAB toolbox (http://www.math.mcgill.ca/keith/surfstat). We tested 5 vertex-wise regression models where (1) cortical thickness at 73 years at each vertex was the dependent variable and WMH volume at 73 years was the independent variable; (2) cortical thickness at 76 years at each vertex was the dependent variable and WMH volume at 76 years was the independent variable; (3) change in cortical thickness at each vertex was the dependent variable and WMH volume at 73 years was the independent variable; (4) cortical thickness at 73 years at each vertex was the dependant variable and change in WMH volume was the independent variable; and (5) change in cortical thickness at each vertex was the dependent variable and change in WMH volume was the independent variable. We defined change in WMH and cortical thickness as individual measurements at 76 years minus measurements at 73 years.

We used false discovery rate to correct for multiple comparisons and calculated *Q* values, that is, false discovery rate–corrected *P* values,^24^ for all vertex-wise regressions thresholded at 0.05. As reported by others,^3^ all models were controlled for sex, MMSE, age in days, years of education, body mass index, and VRF. Finally, we also included childhood (age 11) intelligence quotient as a controlling variable to test whether any associations between WMH and cortical thickness were because of the influence of premorbid levels of cognitive ability.

## Results

### Baseline Only and Follow-Up Subject Comparisons

There were no significant differences at baseline (73 years) between subjects who did return for follow-up cortical thickness measurement and subjects who did not return for follow-up (at 76 years) in overall mean cortical thickness (3.11 mm versus 3.10 mm, *t*=0.75; *P*=0.46); WMH volume (0.78% intracranial volume versus 0.84% intracranial volume, *t*=−0.78; *P*=0.43); history of cardiovascular disease (27.3% versus 26.2%, *z*=0.29; *P*=0.39); current smoking (6.5% versus 8.2%, *z*=−0.76; *P*=0.22); hypercholesterolemia (40.0% versus 43.1%, *z*=−0.71; *P*=0.24); hypertension (46.5% versus 51.8%, *z*=−1.2; *P*=0.11); diabetes mellitus (10.1% versus 11.3%, *z*=−0.43; *P*=0.33); body mass index (27.8 versus 27.8, *t*=−0.21; *P*=0.84); years of education (10.85 years versus 10.89 years, *t*=−0.448; *P*= 0.65); nor age 11 intelligence quotient (101.9 versus 100.2, *t*=1.24; *P*=0.22). However, subjects who did return had higher MMSE scores at baseline than those who did not return (28.9 versus 28.6, *t*=2.49; *P*=0.01).

The remaining results are only for the 351 longitudinal subjects who had full clinical, cognitive, and brain MRI data at baseline and follow-up.

### Baseline, Follow-Up, and Changes in Cognitive, VRF, Cortical Thickness, and WMH Measurements

Baseline, follow-up, and changes in cognitive, VRF, cortical thickness, and WMH measurements are shown in Table 1. There were significant increases in the proportions of subjects with hypertension, hypercholesterolemia, and cardiovascular disease and a decrease in MMSE from baseline (73 years) to follow-up (76 years). Overall, mean cortical thickness generally decreased (Cohen’s *d* of change=−0.45) with age, and WMH volume generally increased (Cohen’s *d* of change=0.93) with age (Table 1). The Spearman correlation matrix between all brain changes and independent variables (Table 2) shows that cortex thinning was generally more pronounced in older subjects (*ρ*=−0.13; *P*=0.02). All other independent variables had limited partial effects on WMH and cortical thinning (beyond the effect of time point; Table 2).

**Table 1.**
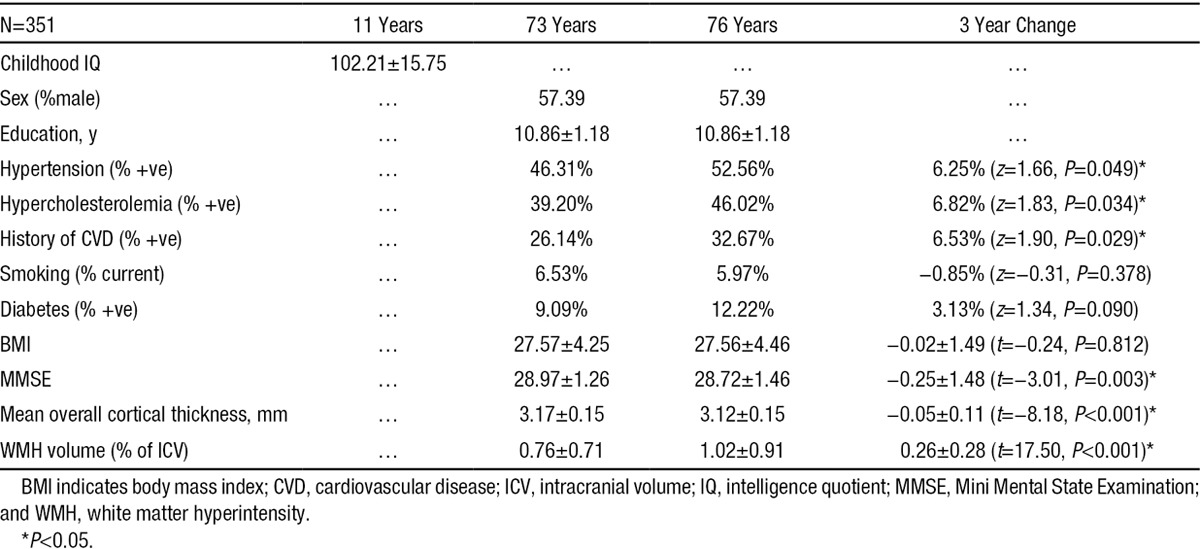
Baseline, Follow-Up, and Changes in Cognitive, VRF, Cortical Thickness, and WMH Measurements

**Table 2.**
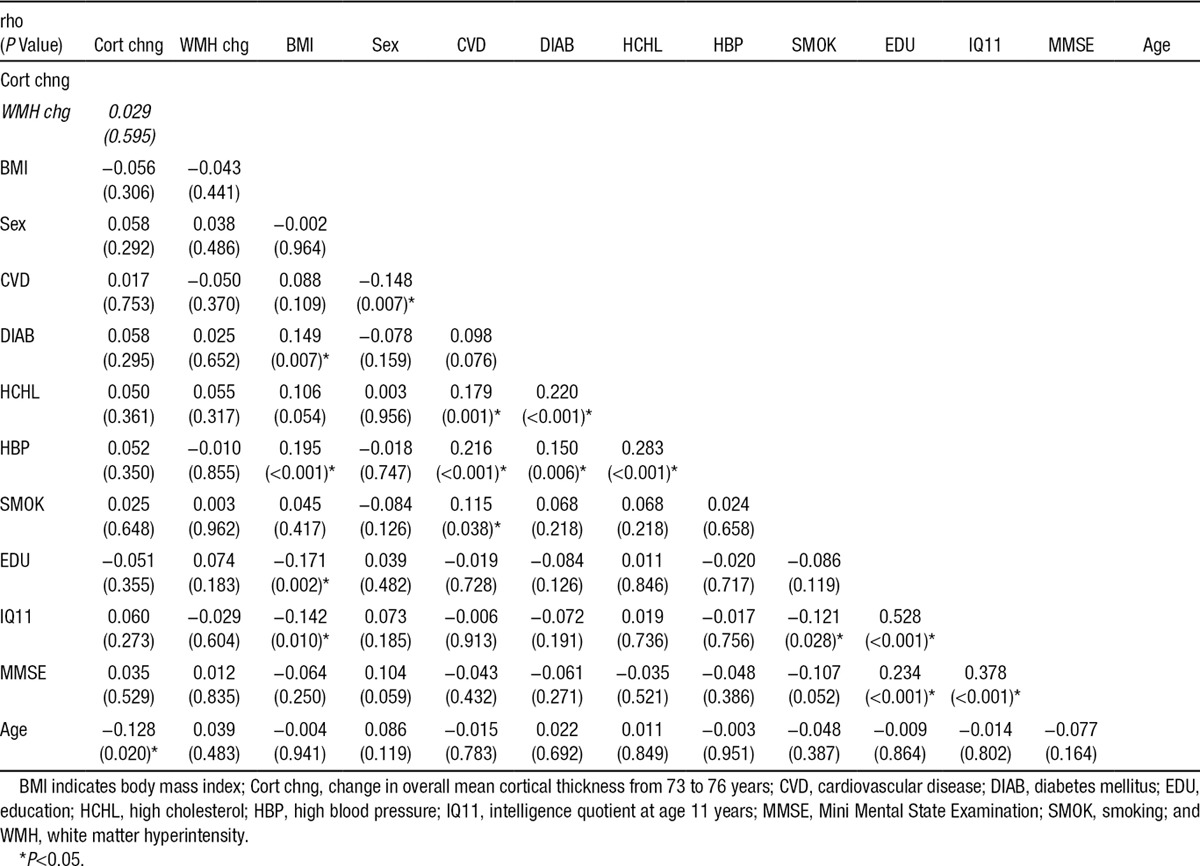
Spearman Correlation Matrix of Overall Mean Cortical Thickness and WMH Changes and Independent Variables

### Cross-Sectional and Longitudinal Global Correlations Between Overall Mean Cortical Thickness and WMH Volume

Cross-sectional global correlations between overall mean cortical thickness and WMH volume at 73 years (*r*=−0.06; *P*=0.27) and 76 years (*r*=−0.08; *P*=0.12) were not significant. Pairwise longitudinal global correlations between (1) WMH volume at 73 years and change in overall mean cortical thickness (*r*=−0.07; *P*=0.19); (2) overall mean cortical thickness at 73 years and change in WMH volume (*r*=0.01; *P*= 0.82); and (3) change in WMH volume and change in overall mean cortical thickness (*r*=−0.02; *P*=0.67) were also not significant.

### Cross-Sectional Vertex-Wise Regression Models of Regional Cortical Thickness and WMH Volume

**Figure 2.**
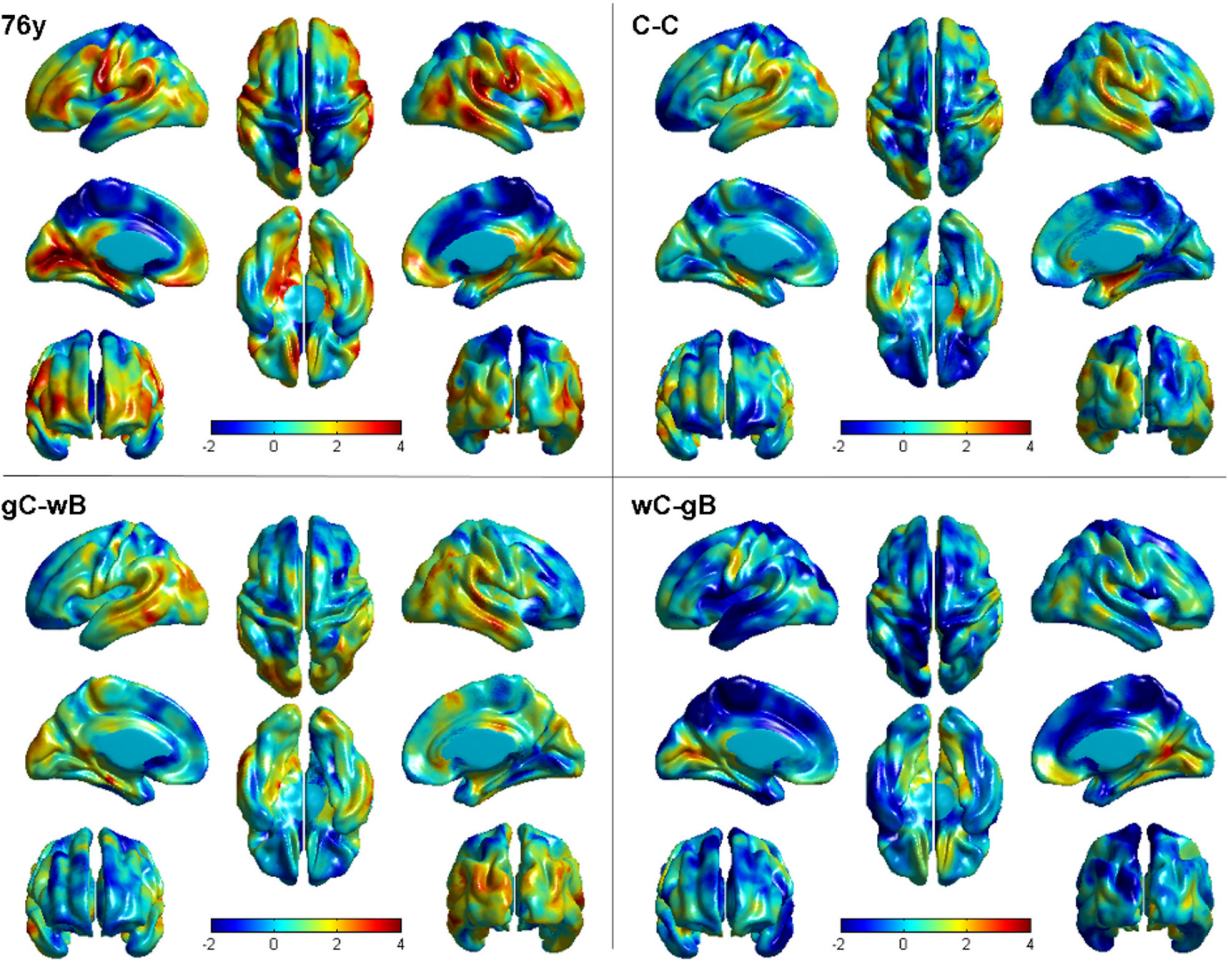
Cross-sectional (76y panel) and longitudinal (C-C, gC-wB, and wC-gB panels) *t*-maps of vertex-wise associations between cortical thickness and whole white matter hyperintensity (WMH) volume. Warm colors show where greater WMH volume is associated with reduced cortical thickness. The significance of these associations is shown in Figure 3. C-C indicates cortical thickness change and WMH volume change from 73 to 76 years; gC-wB, cortical thickness change and WMH volume at 73 years; wC-gB, WMH volume change and cortical thickness at 73 years.

Cross-sectional vertex-wise regression models of cortical thickness and WMH volume at 76 years are shown in Figure 2. Cross-sectional data from 73 years are not shown because the pattern of associations between cortical thickness and WMH volume was almost identical at 73 and 76 years. All models are corrected for VRF, MMSE, education level, and sex. Inclusion of age 11 intelligence quotient as a controlling variable made little difference to the cortical *t*-maps (data not shown).

**Figure 3.**
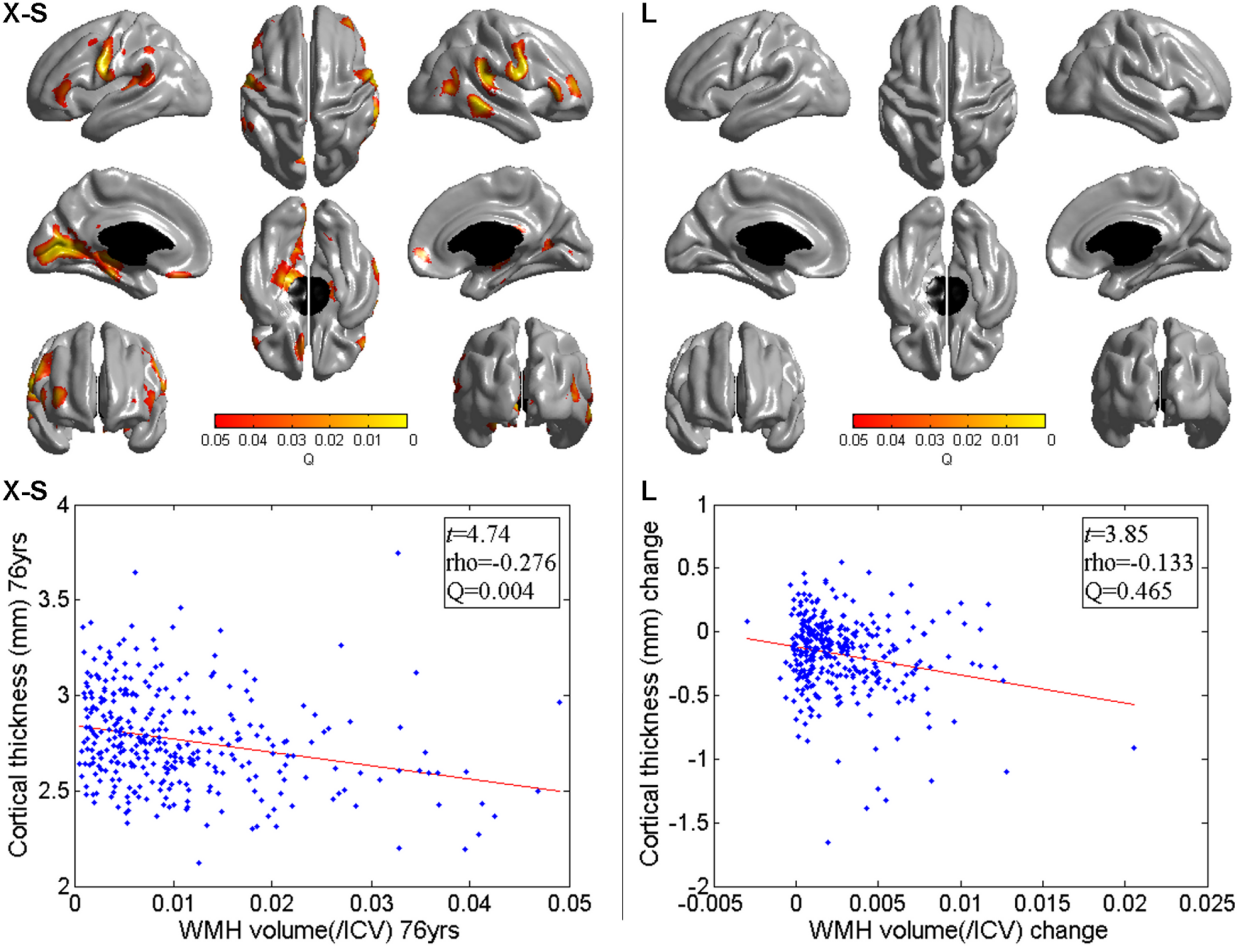
Significance of cross-sectional (X-S) and longitudinal (L) vertex-wise associations between cortical thickness and white matter hyperintensity (WMH) volume. There were consistent patterns of negative cross-sectional associations at 73 (data not shown) and 76 years within and surrounding the Sylvian fissure extending superiorly to the parietal lobe, posteriorly to the occipital lobe, and anteriorly to the frontal lobe (**left**). The clear grey Q-map (**right**) shows that no longitudinal associations between cortical thickness and WMH volume were significant.

Warm colors in Figure 2 show regions where greater WMH volume was associated with reduced cortical thickness. The significance of cross-sectional associations is shown on the left panel of Figure 3. There were consistent patterns of negative cross-sectional associations at 73 and 76 years within and surrounding the Sylvian fissure extending superiorly to the parietal lobe, posteriorly to the occipital lobe, and anteriorly to the frontal lobe. Therefore, having greater WMH volume was cross-sectionally associated with reduced cortical thickness in specific regions only, that is, within and surrounding the Sylvian fissure. Associations between greater WMH volume and greater cortical thickness in superior regions (cold colors in Figure 2) were all nonsignificant.

A scatter plot of the peak cross-sectional association in the Sylvian fissure and surrounding area at 76 years (*ρ*=−0.276; *Q*=0.004) is shown in Figure 3.

### Longitudinal Vertex-Wise Regression Models of Regional Cortical Thickness and WMH Volume

Longitudinal vertex-wise associations between (1) baseline WMH volume and change in cortical thickness (gC-wB in Figure 2); (2) baseline cortical thickness and change in WMH volume (wC-gB in Figure 2); and (3) change in WMH volume and change in cortical thickness (C-C in Figure 2) were all nonsignificant across the cortex (*Q*>0.05; Figure 3). Therefore, having a larger WMH volume at 73 years (or larger change in WMH volume between 73 and 76 years) did not predict greater cortical thinning between 73 and 76 years at any part of the cortex. Neither did a thinner cortex at 73 years predict greater WMH growth between 73 and 76 years.

The longitudinal association between WMH change and overall mean cortical thickness change was descriptively much stronger in subjects with MMSE≤26 (*r*=−0.220; *P*=0.41 versus *r*=−0.003; *P*=0.96) but this was not statistically significant potentially because of the small number of subjects with MMSE≤26 (N=16).

## Discussion

We have replicated cross-sectional associations between greater WMH volume and regional cortical thinning around the Sylvian fissure^3,5,9–12^; however, we found no longitudinal associations between (1) baseline WMH volume and change in cortical thickness; (2) baseline cortical thickness and change in WMH volume; or (3) change in WMH volume and change in cortical thickness at any part of the cortex in community-dwelling subjects from 73 to 76 years. The cross-sectional associations found here between greater WMH volume and reduced cortical thickness in the region of the Sylvian fissure are consistent with previous GM volume,^10^ voxel-based morphometry,^5,11^ and cortical thickness^3^ studies. As with previous studies, the regions of cortical thinning–WMH associations that we found are not consistent with the most frequent WMH locations and areas of expansion,^4^ for example, centrifugally around the ventricles and superiorly towards the cranial vertex.

Our results suggest that WMH volume and cortical atrophy both worsen with age and that their individual differences share some causes—thus the cross-sectional associations. However, their changes from 73 to 76 years do not appear to be associated, and such correlated change would have been one indicator of a possible causal association. This conclusion is consistent with a longitudinal study in CADASIL patients that, although finding strong associations between lacunar lesions and cortical morphological changes, found a limited association between cortical morphological changes and WMH volume.^13,25^

Strengths of our study include the ability to test longitudinal and cross-sectional associations between WMH volume and regional cortical thickness in a large sample of community-dwelling subjects. Other strengths include the age-homogeneous subjects with childhood intelligence quotient assessments and who are now in the eighth decade of life where the risk of dementia increases substantially.^26^ As well as the narrow age range, other novel features of the LBC1936 study (eg, all subjects are white Caucasian) may have minimized any potentially strong confounding effects that factors such as age, mixed ethnicity, and geography might have had in a less homogeneous sample. We measured WMH volume and cortical thickness using well-validated quantitative techniques that we manually checked and quality controlled post-pipeline for each subject at both time points.^17,20,22^ The raw brain MRI from which we measured WMH volume and cortical thickness were obtained using the same protocol on the same carefully maintained scanner at both time points.^16^

Despite these strengths and our replication of previous cross-sectional findings,^3,5,10,11^ our study has limitations. The follow-up time of 3 years is a major limitation because it may not have been long enough to detect correlated changes between WMH and cortical thinning. We chose 3-year follow-up (rather than a longer time) to maximize subject retention and to be consistent with previous studies, for example, Austrian Stroke Prevention Study.^1,27^ Further, we have previously detected cross-sectional differences in WMH because of age within the narrow age band (<3 years) in the LBC1936 study.^28^ We are studying these subjects again at 6 years follow-up, and this may provide better evidence for any potentially causal relationships not identified here. We will attempt to ascertain the reasons for subjects lost to follow-up and will use full information maximum likelihood analyses, checked against analyses of completers, to minimize the effect of loss to follow-up. We defined change as individual measurements at 76 years minus measurements at 73 years. We are aware that there are other ways of assessing change, for example, those often applied to cognitive variable change.^29^ However, the approach we used here is often applied to measure changes in brain morphology.^30^ Although the homogeneous nature of the LBC1936 cohort may provide increased power from having less need to control for confounding variables, for example, age and ethnicity, it limits the generalizability of our results. The longitudinal subjects we assessed here generally had higher MMSE than subjects who did not return for follow-up, and this may also limit the generalizability of our results, for example, longitudinal associations between WMH, and cortical thinning may be stronger in subjects with lower cognitive scores. We could not adequately test this here because of the small number of subjects with MMSE≤26 (N=16), and future work is required to determine whether associations are stronger in cognitively impaired subjects. Although the locations of cross-sectional associations that we (and others^3,5,9–12^) found between WMH and cortical thinning do not directly reflect common areas for WMH expansion, areas of associations were proximate to the tapetum of the corpus callosum fiber tracts which extend inferiorly and anteriorly into the temporal lobes.^31^ Further work is required to determine whether the locations of cross-sectional WMH and cortical thinning associations are because of an indirect connection through the perisylvian cortex and tapetum of the corpus callosum. Finally, it is difficult to prove or disprove a causal relationship between WMH and cortical thinning in observational studies. However, we adjusted for a number of variables known to influence WMH and cortical thinning, and although correlation is not necessarily causation, correlation is fundamental to causation.^32,33^ Therefore, the lack of longitudinal correlations implies the lack of a causal relationship from 73 to 76 years.

Notwithstanding these limitations, we have shown that although they both worsen with age, WMH volume progression and regional cortical thinning do not seem to have a correlative/causal longitudinal relationship from 73 to 76 years. Further longitudinal studies with longer follow-up times and with more time points at different ages are required to determine whether causal relationships become apparent over longer periods of time and/or at different stages of life. The underlying cause(s) of WMH growth and cortical thinning have yet to be fully determined.

## Acknowledgments

We thank the funders, participants, research centers, clinical, and administrative staff who contributed to the LBC1936 study (detailed fully at http://www.lothianbirthcohort.ed.ac.uk/).

## Sources of Funding

This work was funded by a Scottish Funding Council Early Career Researcher grant to the Scottish Imaging Network—A Platform for Scientific Excellence (http://www.sinapse.ac.uk; DAD); Research into Ageing program grant (Drs Deary and Starr) and the Age UK-funded Disconnected Mind project (Drs Deary, Starr, and Wardlaw), with additional funding from the UK Medical Research Council (Drs Deary, Starr, and Wardlaw, and M.E. Bastin); and Scottish Funding Council through the Scottish Imaging Network—A Platform for Scientific Excellence (Dr Wardlaw).

## Disclosures

Dr Wardlaw reports money (grants) paid to The University of Edinburgh from Medical Research Council, Age UK, Row Fogo Charitable Trust, and Scottish Funding Council for her efforts on the LBC1936 study and various imaging projects. Dr Deary reports money (grants) paid to The University of Edinburgh from Medical Research Council and Age UK for his efforts on the LBC1936 study. Dr Deary reports money paid to him for board membership on Medical Research Council. All other authors have no disclosures.
